# Synthesis and structure–activity relationship studies of N-terminal analogues of the lipopeptide antibiotics brevicidine and laterocidine†

**DOI:** 10.1039/d2md00281g

**Published:** 2022-10-27

**Authors:** Ross D. Ballantine, Karol Al Ayed, Samantha J. Bann, Michael Hoekstra, Nathaniel I. Martin, Stephen A. Cochrane

**Affiliations:** School of Chemistry and Chemical Engineering, David Keir Building, Queen's University Belfast Stranmillis Road Belfast BT9 5AG UK s.cochrane@qub.ac.uk; Biological Chemistry Group, Institute of Biology, Leiden University Sylviusweg 72 2333 BE Leiden The Netherlands

## Abstract

The brevicidine and laterocidine family of lipopeptide antibiotics exhibit strong activity against multidrug-resistant Gram-negative bacteria, while showing low propensity to induce resistance. Both peptides feature a branched lipid tail on the N-terminal residue, which for brevicidine is chiral. Here, we report the synthesis and biological evaluation of a library of brevicidine and laterocidine analogues wherein the N-terminal lipid is replaced with linear achiral fatty acids. Optimal lipid chain lengths were determined and new analogues with strong activity against colistin-resistant *E. coli* produced.

## Abstract 2

SAR studies on brevicidine and laterocidine yield new analogues with strong activity against multidrug-resistant Gram-negative bacteria.

## Graphical abstract

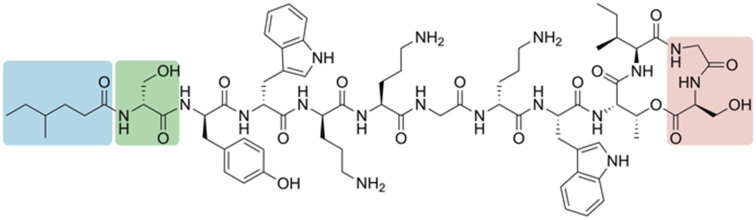

## Introduction

Antimicrobial resistance (AMR) is set to become a major crisis within our lifetime. In addition to the economic costs, it is estimated that the number of deaths caused by AMR will rise to 10 million annually by 2050.^1^ In fact, the number of deaths attributable to bacterial AMR surpassed the yearly deaths caused by breast cancer in 2019.^2^ Given the pressing need for new antibacterial agents, synthesis and structure–activity relationship (SAR) studies with novel lead compounds remain valuable strategies for addressing the rising tide of AMR.

Non-ribosomal lipopeptides represent a gold mine of potential antimicrobials with desirable therapeutic advantages, including strong activity against multidrug-resistant bacteria, multi-faceted modes of action,^3^ and superior proteolytic stability when compared to ribosomal antimicrobial peptides.^4–7^ Their superior stability arises from the presence of d-amino acids and/or macrocyclic motifs, both of which improve proteolytic stability. Lipopeptides are secondary metabolites produced by non-ribosomal peptide synthetases (NRPSs), and are often *N*-acylated with a lipid tail.^8^ The lipids are biosynthetically derived from the branched amino acids (valine, leucine and isoleucine), therefore it is common that bacterial lipopeptides feature a similarly branched acyl group.^9^ Owing to the synthetic challenge and expense associated with incorporating these features into peptide synthesis, a common focus of SAR studies is to vary the N-terminal lipid tail. Lipid tail libraries have been created for many lipopeptides, including tridecaptins,^10^ paenibacterin,^7^ cerexins^11^ and polymyxins.^12^

**Fig. 1:**
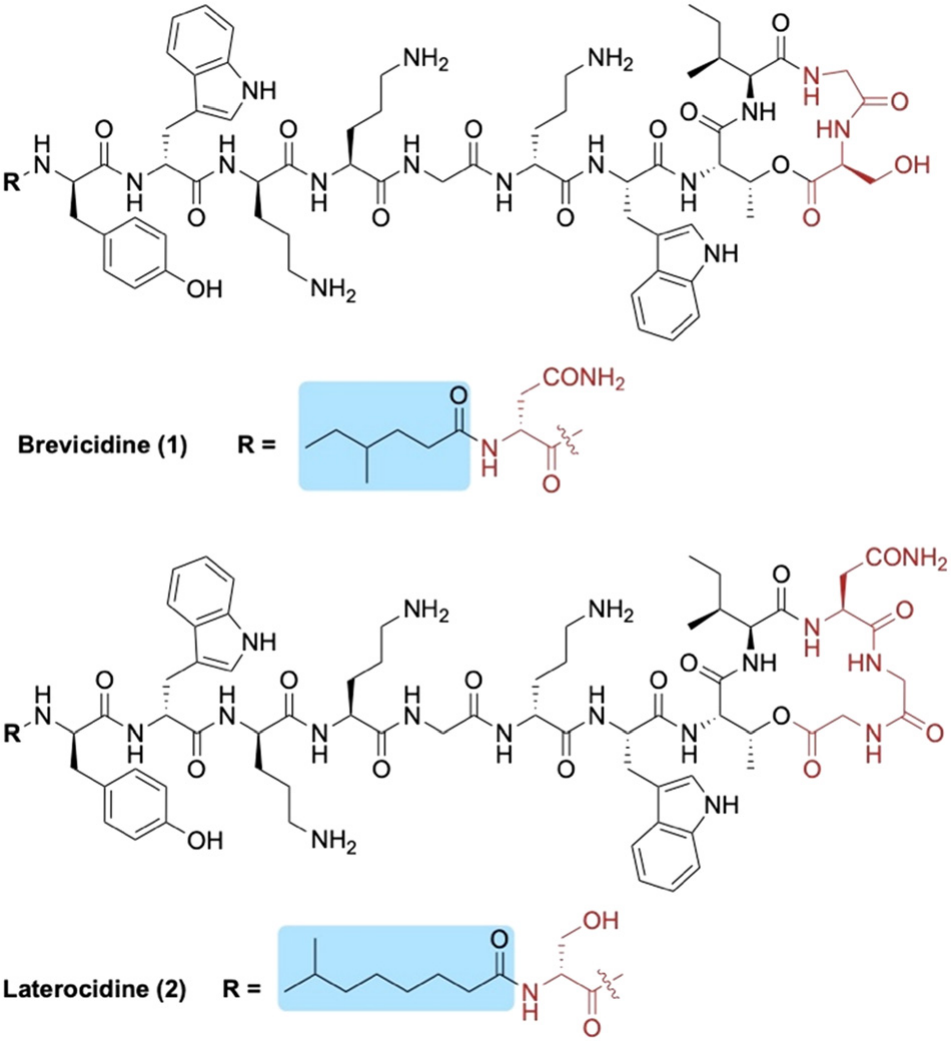
Structures of brevicidine (1) and laterocidine (2). N-terminal lipid tails are highlighted in blue and structural differences between the two lipopeptides in red.

Brevicidine (1) and laterocidine (2) are two novel peptides that were recently reported by Li *et al.* following a biosynthetic gene cluster mining strategy (Fig. 1).^13^ Given their strong antimicrobial activity against Gram-negative bacteria (including colistin-resistant *E. coli*), along with their low cytotoxicity and low propensity to induce resistance, we recently developed methods to access both brevicidine and laterocidine by solid-phase peptide synthesis (SPPS).^14^ The ability to synthesize this family of lipopeptides has allowed for the possibility of SAR studies including the structurally related relacidines.^15^ Brevicidine (1) and laterocidine (2) each feature an N-terminal acyl chain; 4-methylhexanoyl in the former and 6-methyloctanoyl in the latter. Herein, we report the development of novel N-terminal lipid analogues of brevicidine and laterocidine with strong and selective activity against Gram-negative bacteria.

## Results and discussion

**Scheme 1:**
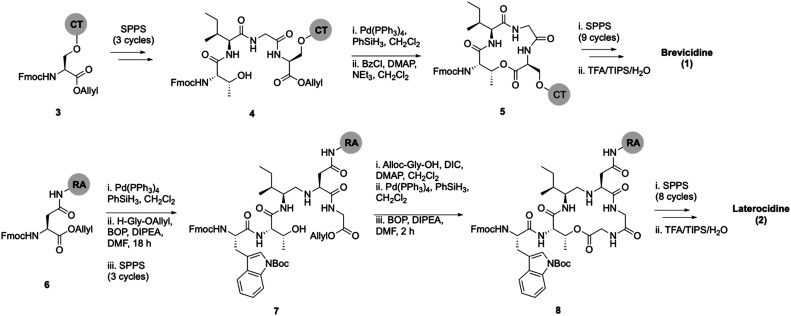
On-resin syntheses of brevicidine (1) (top) laterocidine (2) (bottom). CT: 2-chlorotrityl chloride resin, RA: rink amide resin.

Lipid analogues were synthesized following our previously reported methods (Scheme 1).^14^ Briefly: for analogues of brevicidine, Fmoc-Ser-OAllyl was first loaded on to 2-chlorotrityl (CT) chloride resin *via* the side chain and standard Fmoc-SPPS was performed to synthesize the tetrapeptide (4). Allyl ester deprotection, followed by an on-resin modified Yamaguchi esterification afforded the macrocyclic lactone portion of the peptide, which was subsequently extended through the N-terminus *via* SPPS to obtain the desired analogues. Similarly, for analogues of laterocidine, Fmoc-Asp-OAllyl was first loaded onto rink amide (RA) resin *via* its side chain. The allyl group was next removed and H-Gly-OAllyl was coupled after which SPPS was used to obtain linear pentapeptide (7). An on-resin Steglich esterification between the free hydroxyl of threonine and Alloc-Gly-OH was then performed. Both allyl and Alloc groups were subsequently removed, followed by an on-resin macrolactamization which yielded the laterocidine macrocycle. The cyclic intermediate was then further elaborated through to the N-terminus by SPPS. Natural brevicidine has a chiral 4-methylhexanoyl lipid tail, the configuration of which has not been previously reported. This likely has an (*S*)-configuration as such lipids are often derived from isoleucine.^8^ As chiral lipids are expensive and/or must be chemically synthesized, we chose to synthesise lipid tail analogues containing cheaper, commercially available lipids. The brevicidine and laterocidine variants prepared included unacylated peptides (9 & 18) and C2–C16 lipidated brevicidine (10–17) and laterocidine (19–26) analogues, with lipid length incrementally increasing by two carbons for each analogue. Peptides were synthesized in overall yields ranging between 5–27% (after HPLC purification).

The minimum inhibitory concentrations (MICs) of brevicidine and laterocidine analogues were determined against a panel of clinically relevant ESKAPE pathogens; *Escherichia coli* (*E. coli*), *Klebsiella pneumoniae* (*K. pneumoniae*), *Acinetobacter baumannii* (*A. baumannii*), *Pseudomonas aeruginosa* (*P. aeuroginosa*) and *Staphylococcus aureus* (*S. aureus*), which cause the majority of nosocomial infections in the United States.^16^ Notably, while colistin (polymyxin E) is used as a last-resort antibiotic in the treatment of infections caused by many Gram-negative bacteria, the emergence of plasmid-borne genes conferring colistin resistance (*mcr*) threatens to render this drug ineffectual.^17^ In this resistance mechanism, the polymyxin target (lipid A) is modified, reducing binding affinity. For this reason, a strain of colistin-resistant *E. coli* carrying the *mcr*-1 gene was also included in the panel.

For the brevicidine lipid analogues, H-Brev (9), C2-Brev (10) and C16-Brev (17) showed complete ablation of antimicrobial activity (>32 μg mL^−1^) (Table 1). The latter could be due to reduced solubility of the peptide in Mueller Hinton broth (MHB), despite using DMSO as an additive, or activity could be diminished by hydrophobic self-aggregation interfering with the peptide's ability to interact with the bacterial membrane. Inversely, peptides lacking a lipid tail or with a very short lipid are likely unable to insert into the bacterial membrane, thus limiting their ability to disrupt the membrane or self-permeabilise through to the periplasm.^18^ A similar observation has been made for unacylated analogues of polymyxin B and colistin, which display no antimicrobial activity, despite retaining the ability to efficiently bind with high specificity to lipopolysaccharide (LPS).^18^ Notably, our lipid scan with brevicidine and laterocidine revealed an apparent double “sweet-spot” in activity with C6-Brev (12), which generally showed a two-fold decrease in activity across strains, and C10-Brev (14) which maintained comparable activity to Brevicidine (1). C10-Brev (14) likely has similar hydrophobic properties to the natural branched C7 lipid in Brev, whereas C8-Brev (13) is less hydrophobic and less active. The higher activity of C6 *vs.* C8 was unexpected but could be due to improved solubility. In the case of the laterocidine analogues, a broader increase in antimicrobial activity was observed for analogues 20–25 with C8-Lat (22) and C10-Lat (23) exhibiting the same activity as laterocidine (2).

**Table 1:** Antimicrobial activity of brevicidine and laterocidine analogues 9–26

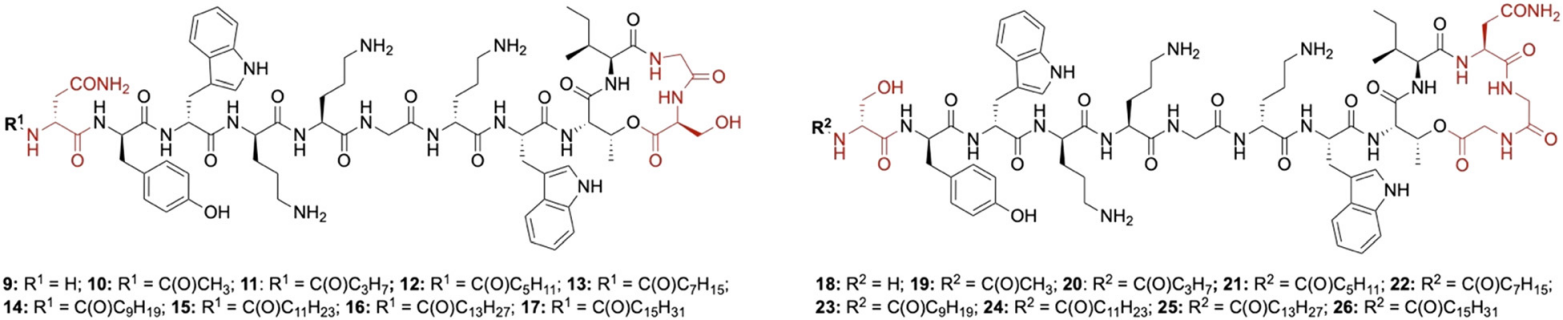								
Compound	Lipid chain length	Antimicrobial activity (μg mL^−1^)						% Hemolytic activity
		*E. coli* ATCC 25922	*E. coli* ATCC 25922 MCR-1	*K. pneumoniae* ATCC 13883	*A. baumannii* ATCC 17961	*P. aeruginosa* PAO1	*S. aureus* USA300	Sheep red blood cells
1	C7	4	4	2	4	8	>32	0.8
9	H	>32	>32	>32	>32	16	>32	0.2
10	C2	>32	>32	>32	>32	8	>32	0.2
11	C4	32	32	8	32	16	>32	0.1
12	C6	8	8	4	16	>32	>32	0.3
13	C8	16	16	16	16	8	>32	0.4
14	C10	4	4	4	4	8	32	6.2
15	C12	8	8	8	4	16	32	13.3
16	C14	16	16	32	4	32	>32	18.0
17	C16	>32	>32	>32	32	>32	>32	13.3
2	C9	2	2	2–4	2	4	>32	0.4
18	H	>32	>32	>32	>32	>32	>32	0.2
19	C2	>32	>32	>32	>32	16	>32	0.2
20	C4	16	32	32	>32	8	>32	0.3
21	C6	4	8	8	16	4	>32	0.2
22	C8	2	2	2	4	2	>32	0.2
23	C10	2	2	2–4	2	2	>32	2.1
24	C12	4	4	8	2	8	32	21.5
25	C14	8	8	8	2	8	16	56.2
26	C16	32	32	>32	4	32	32	46.1
Colistin	C8/C9	0.5	8	0.5	≤0.25	4	>32	<0.1%
0.1% TX100	ND	ND	ND	ND	ND	ND	ND	100

H-Lat (18), C2-Lat (19) and C16-Lat (26) showed a marked decrease in activity – with the exception of C16-Lat (26) against *A. baumannii* (4 μg mL^−1^). The more hydrophobic analogues are likely less active due to their poorer solubility in aqueous media. Gratifyingly, the activities of the brevicidine and laterocidine analogues against *E. coli* were unaffected by the presence of the *mcr*-1 resistance gene, paralleling the early *in vitro* results by Li *et al.*^13^ These findings further underscore the potential for this class of lipopeptide antibiotics to be developed as a therapeutic alternative against drug-resistant infections.

Having ascertained the antimicrobial activity of all synthetic peptides against a panel of ESKAPE pathogens, we next assessed their mammalian toxicity with hemolytic assays using sheep red blood cells. The % hemolysis for all peptides at 64 μg mL^−1^ was determined, with the hemolysis induced by the surfactant 0.1% Triton X-100 taken as 100%. The peptide concentration used is 32× the MIC of the most potent analogues. Hemolysis was <1% for all analogues with a C8 chain or shorter, including the strongest antibacterial peptide C8-Lat (22). Hemolytic activity increased up to C14 (>50% for C14-Lat) and then decreased at longer chain lengths, perhaps due to decreased solubility of peptides or aggregation.

In summary, a library of *N*-terminal lipid analogues was generated for brevicidine (9–17) and laterocidine (18–26) using our previously established synthetic approaches. The peptides were assayed *in vitro* against a panel of ESKAPE pathogens to identify analogues with comparable activities to synthetic brevicidine (1) and laterocidine (2). The substitution with a decanoyl tail in both brevicidine (14) and laterocidine (23) had no effect on the antimicrobial activity, including colistin-resistant *E. coli*. This strong activity against drug-resistant Gram-negative bacteria, coupled with the reduced synthetic cost, highlights these analogues as potential therapeutic candidates for future development.

## Conflicts of interest

There are no conflicts to declare.

## Supplementary Material

MD-013-D2MD00281G-s001
