# Supplementary material for: Synthesis and structure–activity relationship studies of N-terminal analogues of the lipopeptide antibiotics brevicidine and laterocidine
Source: RSC Med Chem. 2022 Oct 27;13(12):1640–3. doi: 10.1039/d2md00281g (PMC9749936; doi:10.1039/d2md00281g)
Supplement: MD-013-D2MD00281G-s001 [file MD-013-D2MD00281G-s001.pdf]

Electronic Supplementary Material (ESI) for RSC Medicinal Chemistry.
This journal is © The Royal Society of Chemistry 2022

Supplementary Information

FOR

Synthesis and Structure-Activity Relationship Studies of N-Terminal Analogues of the Lipopeptide Antibiotics Brevicidine and Laterocidine

Ross D. Ballantine,[‡a] Karol Al Ayed,[‡b] Samantha J. Bann,[a] Michael Hoekstra,[b] Nathaniel I. Martin*[b] and Stephen A. Cochrane*[a]

[a] School of Chemistry and Chemical Engineering, David Keir Building, Stranmillis Road, Queen's University Belfast, Belfast, UK, BT9 5AG; [b] Biological Chemistry Group, Institute of Biology, Leiden University, Sylviusweg 72, 2333 BE, Leiden, The Netherlands.

‡Equal contribution
*To whom correspondence should be addressed. Email: s.cochrane@qub.ac.uk

TABLE OF CONTENTS

Section	Contents	Page Number
I	Materials	S2
II	General procedure for manual peptide synthesis	S2
III	Purification and analysis of peptides	S4
IV	Antimicrobial testing	S5
V	Hemolytic assays	S6
VI	HPLC and HRMS analysis of peptides	S7
VII	References	S18

I. Materials

Brevicidine Analogues. All proteinogenic Fmoc-amino acids used in this study were purchased from CEM. The remaining Fmoc-amino acids, including Fmoc-D-Trp(Boc)-OH, Fmoc-D-Tyr(tBu)-OH and Fmoc-D-Asn(Trt)-OH were purchased from Fluorochem. Fmoc-D-Orn(Boc)-OH was purchased from Merck. Fmoc-L-Orn(Boc)-OH was purchased from ChemImpex. Fmoc-Ser-OAllyl,[1] Fmoc-Asp-OAllyl,[2] TFA·H_2N-Gly-OAllyl[3] were synthesized according to referenced literature procedures. Acetic anhydride, butyric acid, decanoic acid and dodecanoic acid were purchased from Alfa Aesar. Hexanoic acid and octanoic acid were purchased from Sigma-Aldrich. Myristic acid was sourced from the C6 to C14 Fatty Acid Kit by Matreya Inc. Palmitic acid was purchased from Fluorochem. [Bis(dimethylamino)methylene]-1*H*-1,2,3-triazolo[4,5-*b*]pyridinium-3-oxidhexafluorophosphate (HATU), trifluoroacetic acid (TFA) and triisopropylsilane (TIPS) were also purchased from Fluorochem. 2-Chlorotrityl chloride resin and diisopropylethylamine (DIPEA) were purchased from Sigma-Aldrich. HPLC grade acetonitrile (MeCN), dichloromethane (CH_2Cl_2) and dimethylformamide (DMF) were purchased from Merck. All chemicals were used without further purification.

Laterocidine analogues. Fmoc-L-Orn(Boc)-OH and Fmoc-D-Orn(Boc)-OH were purchased from Combi-Blocks. All other Fmoc-amino acids and the Rink amide MBHA resin were purchased from P3 BioSystems. Acetic anhydride, butyric acid, lauric acid, myristic acid, palmitic acid, sodium diethyldithiocarbamate trihydrate and pyridine were purchased from Acros Organics. Hexanoic acid, octanoic acid and decanoic acid were purchased from Alfa Aesar. ((1H-Benzo[d][1,2,3]triazol-1-yl)oxy)tris(dimethylamino)phosphonium hexafluorophosphate (BOP), N,N-Diisopropylcarbodiimide (DIC) and triisopropylsilane (TIPS) were purchased from Manchester Organics. 4-Dimethylaminopyridine (DMAP) was purchased from Sigma Aldrich. Diisopropylethylamine (DIPEA), piperidine, trifluoroacetic acid (TFA) and dimethyl sulfoxide (DMSO) were purchased from Carl Roth. Dichloromethane (CH_2Cl_2) and petroleum ether were purchased from VWR Chemicals. Acetonitrile (MeCN), dimethylformamide (DMF) and methyl tertiary-butyl ether (MTBE) were purchased from Biosolve. Tetrakis(triphenylphosphine)palladium(0) and phenylsilane were purchased from Fluorochem.

Antimicrobial testing

Colisitin sulfate was purchased from Activate Scientific. Kanamycin monosulfate was purchased from MP Biomedicals. *E. coli* ATCC 25922, *S. aureus* USA300 (ATCC BAA1717), *K. pneumoniae* ATCC 13883 and *A. baumannii* ATCC 17961 belong to the American Type Culture Collection (ATCC). P. aeruginosa PAO1 was kindly provided by L.H.C. Quarles Van Ufford, Utrecht University, Utrecht, The Netherlands. *E. coli* ATCC 25922 MCR-1 was transfected in house using the pGDP2-MCR1 plasmid kindly provided by Yong-Xin Li, The University of Hong Kong, Hong Kong, China. Sheep blood agar plates (Ref. PB5039A) were purchased from Thermo Scientific. Tryptic soy broth (Ref. 02-200-500) was purchased from Scharlab. Mueller-Hinton broth (Ref. X927.1) was purchased from Carl Roth. Polypropylene 96-wells plates (Ref. 3879) were purchased from Corning.

II. General procedure for manual peptide synthesis

Brevicidine analogues. Analogues of brevicidine were synthesized according to our previously reported synthesis.[4] To a flame dried 50 mL round bottom flask was added Fmoc-Ser-OAllyl (220 mg, 0.600 mmol) and dry dichloromethane (CH_2Cl_2) (10.0 mL). 2-Chlorotrityl (CT) chloride resin (2.00 g, 0.81 mmol g^{-1}) and DIPEA (420 µL, 2.40 mmol) were added. The suspension was stirred under argon for 24 h at 45 °C, after which the resin was filtered through a manual SPPS vessel and

washed with CH_2Cl_2 (3 x 10 mL). The resin was then capped by adding a solution of MeOH, DIPEA and CH_2Cl_2 (20 mL, 10 : 5 : 85) and bubbled with argon for 1 h. The resin was washed with DMF (3 x 10 mL) then CH_2Cl_2 (3 x 10 mL) and dried under a positive pressure of argon. A small portion of resin was then used to ascertain the loading. Estimation of loading level of first residue onto resin (0.16 mmol g^{-1}) was calculated via an Fmoc loading test, as described by Gude *et al*.[5]

Standard Fmoc SPPS protocol was used to extend the peptide to the linear Fmoc-Thr-Ile-Gly-Ser stage. Specifically, loaded resin (1.56 g, 0.25 mmol) was added to a manual SPPS vessel and bubbled in DMF (15 mL) for 15 min to swell. The solvent was discharged and the resin was bubbled in an Fmoc deprotection solution of 20% piperidine in DMF (3 x 15 mL, 2 x 1 min then 1 x 5 min) with argon. The resin was washed with DMF (3 x 15 mL) and a coupling solution of amino acid (5 equiv), HATU (5 equiv) and DIPEA (10 equiv) in DMF (15 mL) was added. The solution was then bubbled with argon for 1 h, before the solution was discharged and the resin washed with DMF (3 x 15 mL). The process was repeated to obtain on-resin linear Fmoc-tetrapeptide. An allyl deprotection solution of tetrakis(triphenylphosphine) palladium (578 mg, 0.500 mmol) and phenylsilane (308 µL, 2.50 mmol) in CH_2Cl_2 and DMF (1:1, 20 mL) was added. The solution was bubbled with argon for 2 h in darkness, after which the deprotection solution was discharged and the resin was washed with DMF (3 x 15 mL), 0.5% sodium diethyldithiocarbamate solution in DMF (4 x 15 mL), DMF (4 x 15 mL) and CH_2Cl_2 (4 x 15 mL). The resin was dried under argon, then added to a 100 mL flame dried round bottom flask under argon. Dry dichloroethane (50 mL), benzoyl chloride (30 µL, 0.258 mmol), triethylamine (139 µL, 9.97 mmol) and catalytic DMAP (3.00 mg, 24.6 µmol) were added. The mixture was stirred overnight at 60 °C. The reaction was cooled to room temperature before the resin was filtered through a manual SPPS vessel and washed with DMF (3 x 15 mL) and CH_2Cl_2 (3 x 15 mL) and dried under argon. To ascertain a successful macrolactonisation, a small sample was cleaved using 2% TFA solution in CH_2Cl_2 (1 mL). The cleavage cocktail was gently agitated for 1 h and filtered through a glass wool plug. The filtrate was concentrated with a gentle stream of argon before being analyzed by LC-MS. Desired cyclic product was identified ($[M+H]^+$ calculated for $C_{30}H_{36}N_4O_8$ 581.2, found (LC-MS) 581.4. Following this modified Yamaguchi esterification, Fmoc SPPS was continued using the above protocol to complete the linear peptide portion. The resin was then divided and the lipid tails were attached to the *N*-terminus using the same procedure (0.05 mmol scale). Acetylated brevicidine (**11**) was synthesized by adding on-resin peptide (306 mg, 50.0 µmol) to a manual SPPS vessel with DMF (5 mL) to swell for 10 min. The solvent was discharged and a solution of acetic anhydride (240 µL, 2.54 mmol), DIPEA (440 µl, 2.53 mmol) and DMAP (1 crystal) in DMF (5 mL) was added and bubbled for 2.5 h with argon. The resin was then washed with DMF (3 x 5 mL) and CH_2Cl_2 (3 x 5 mL) then dried under argon.

The dried resin was then added to a cleavage cocktail of TFA, TIPS and distilled water (3 mL, 95 : 2.5 : 2.5) and heated to 37 °C for 1 h. The suspension was filtered through a glass wool plug and the filtrate concentrated under vacuum. Diethyl ether was used to precipitate the crude peptide, which was then centrifuged and washed with additional diethyl ether. The suspension was centrifuged and the pellet dissolved in the minimal amount of 1:1 acetonitrile and water solution with 0.1% TFA. The crude mixture was subsequently purified by RP-HPLC Method A. Fractions were assessed by LC-MS and product containing fractions were pooled, frozen and lyophilized to yield the lipid analogues.

Laterocidine analogues. Rink amide MBHA (5.00 g, 0.67 mmol g^{-1}) was loaded via the sidechain carboxyl group of Fmoc-Asp-OAllyl (2.65 g, 6.70 mmol) with BOP (2.96 g, 6.70 mmol) and DIPEA (2.33 mL, 13.4 mmol) in DMF (150 mL) overnight. The resin was capped with acetic anhydride and pyridine (3 : 2) for 30 min and the resin loading was determined as above (0.50 mmol g^{-1}). Two batches of the loaded resin (1.00 g, 0.500 mmol) were bubbled in tetrakis(triphenylphosphine) palladium

(150 mg, 0.130 mmol) and phenylsilane (1.50 mL, 12.2 mmol) in CH_2Cl_2 (30 mL) with nitrogen for 1 h. The resin was subsequently washed with CH_2Cl_2 (5 x 20 mL), sodium diethyldithiocarbamic acid trihydrate in DMF (5 mg mL^{-1}, 5 x 20 mL) and DMF (5 x 20 mL). TFA·H_2N-Gly-OAllyl (230 mg, 1.00 mmol) was coupled using BOP (442 mg, 0.999 mmol) and DIPEA (350 µL, 2.01 mmol) under nitrogen flow for 1 h. Fmoc SPPS was continued using a similar procedure as above: Fmoc was removed using a 20% piperidine solution (3 x 10 mL, 2 x 2 min then 1 x 10 min). The resin was washed and a coupling solution of amino acid (4 equiv), BOP (4 equiv) and DIPEA (8 equiv) in DMF (10 mL) was added and bubbled with nitrogen for 1 h. The process was repeated to extend the *N*-terminus to include Trp8. The resin was then treated with Alloc-Gly-OH (1.20 g, 7.54 mmol), DIC (1.20 mL, 7.66 mmol) and DMAP (30.0 mg, 0.246 mmol) in CH_2Cl_2 and DMF (16 mL, 3 : 1) under nitrogen for 18 h. Alloc protecting groups were removed following the aforementioned protocol with tetrakis(triphenylphosphine) palladium. Cyclization was then achieved by adding a solution of BOP (442 mg, 0.999 mmol) and DIPEA (350 µL, 2.01 mmol) in DMF (10 mL) and bubbling with nitrogen for 2 h. The remaining linear N-terminal section of the peptide was synthesized using the above SPPS protocol. The two batches of resin were divided and lipid tails were attached as above on a 0.1 mmol scale. Dried resin was subsequently added to cleavage cocktail containing TFA, TIPS and H_2O (5 mL, 95 : 2.5 : 2.5) for 1.5 h. The reaction mixture was then filtered through cotton and precipitated in MTBE and petroleum ether (1 : 1). The resulting precipitate was washed with fresh MTBE and petroleum ether (1 : 1) and lyophilized from *t*-butanol and water (1 : 1). The crude peptide was then purified by RP-HPLC Method B. Pure fractions were pooled and lyophilized.

III. Purification and analysis of peptides

Prep RP-HPLC purification of crude peptides. Brevicidine analogues were purified by Reversed-Phase High Performance Column Chromatography (RP-HPLC). Purification was performed on a Perkin Elmer HPLC system composed of a 200 series binary pump, UV/Vis detector, vacuum degasser, Rheodyne 7725i injector. The system was operated using ThermoFisher Chromeleon 7.2 software. **Method A (Preparative):** Phenomenex Luna C18 column (5 µm, 250 x 21.2 mm) equipped with a 2 mL sample loop. Runs were performed at a flow rate of 10 mL/min with UV detection at 220 nm. Solvent A = 0.1 % TFA in MilliQ water and solvent B = 0.1 % TFA in MeCN. A gradient method for C_0 – C_{12} brevicidine analogues was employed, starting from 5 % B and 95 % A for 5 min, ramping up to 8 % B over 20 min, then ramping up to 20 % B over 15 min, ramping up to 30 % B over 3 min, ramping again up to 95 % B over 4 min, remaining at 95 % B for 3 min, ramping down to 5 % B over 2 min before staying at 5 % B for 5 min. A gradient method for C_{14} - C_{16} brevicidine analogues was employed, starting from 5 % B and 95 % A for 5 min, ramping up to 27 % solvent B over 6 min, then ramping up to 44 % solvent B over 7 min, ramping up again to 78 % solvent B over 9 min, ramping to 95 % solvent B over 3 min, remaining at 95 % B for 2 min before ramping down to 5 % B over 3 min and remaining there for 5 min. **Method B (Preparative):** Purification was performed on a BESTA-Technik system with a Dr. Maisch ReproSil Gold 120 C18 column (10 µm, 25 x 250 mm) and equipped with a ECOM Flash UV detector. Runs were performed at a flow rate of 12 mL/min with UV detection at 214 nm and 254 nm. Solvent A = 0.1% TFA in water/MeCN (95 : 5) and solvent B = 0.1% TFA in water/MeCN (5 : 95). A gradient method was employed, starting at 100 % solvent A for 5 min, ramping up to 70 % solvent B over 50 min, remaining at 70 % solvent B for 3 min before ramping down to 100 % solvent A over 1 min and remaining there for 5 min. Product containing fractions were pooled, partially concentrated under vacuum, frozen and then lyophilized to yield pure peptides as white flocculent solids. A small amount of purified peptide was analyzed by analytical HPLC. **Method C (Analytical):** Analytical runs of brevicidine analogues were

performed on a Perkin Elmer HPLC system composed of a 200 series quaternary pump, UV/Vis detector, vacuum degasser, Rheodyne 7725i injector equipped with a 200 µL sample loop and Phenomenex Luna C18 column (5 µm, 150 x 4.6 mm). The system was operated using ThermoFisher Chromeleon 7.2 software. Runs were performed at a flow rate of 2 mL/min with UV detection at 220 nm. Solvent A = 0.1 % TFA in MilliQ water and solvent B = 0.1 % TFA in MeCN. A gradient method was employed, starting from 20 % B and 80 % A for 2 min, ramping up to 95 % B over 18 min, ramping down to 20 % B over 0.1 min before staying at 20 % B for 3.9 min. **Method D (Analytical):** Analytical runs of laterocidine analogues were performed on a Shimadzu Prominence-i LC-2030 system with a Dr. Maisch ReproSil Gold 120 C18 (5 µm, 4.6 x 250 mm) at 30 °C. Runs were performed at a flow rate of 1 mL/min with UV detection at 214 nm and 254 nm. Solvent A = 0.1% TFA in water/CAN (95 : 5) and solvent B = 0.1% TFA in water/CAN (5 : 95). A gradient method for C_0 - C_{12} laterocidine was employed, starting at 100% solvent A for 2 min, ramping up to 50 % Solvent B over 45 min, ramping up to 100% solvent B over 1 min, remaining at 100% solvent B for 6 min before ramping down to 100% solvent A over 1 min and remaining there for 5 min. A gradient method for C_{14} - C_{16} laterocidine was employed, starting at 100% solvent A for 2 min, ramping up to 70 % solvent B over 45 min, ramping up to 100% solvent B over 1 min, remaining there for 6 min before ramping down to 100% solvent A and remaining there for 5 min. High resolution mass spectrometry (HRMS) spectra of brevicidine peptides were recorded by Analytical Services and Environmental Projects (ASEP) at Queen's University Belfast on a Waters LCT Premier ToF mass spectrometer using the electrospray ionisation (ESI) technique. HRMS spectra of laterocidine peptides were performed on a Thermo Scientific Dionex UltiMate 3000 HPLC system with a Phenomenex Kinetex C18 (2.6 µm, 2.1 x 150 mm) column at 35 °C and equipped with a diode array detector. The following solvent system, at a flow rate of 0.3 mL/min, was used: solvent A = 0.1% formic acid in water, solvent B = 0.1% formic acid in acetonitrile. A gradient method was employed, starting at 95 % solvent A and 5 % solvent B for 1 min, ramping up to 95 % solvent B over 9 min, ramping up to 98 % solvent B over 1 min, remaining there for 1 min before ramping back down to 95 % solvent A over 2 min and remaining there for 1 min. The system was connected to a Bruker micrOTOF-Q II mass spectrometer (ESI ionization) calibrated internally with sodium formate.

IV. Antimicrobial testing

All minimum inhibitory concentrations (MICs) were determined according to Clinical and Standards Laboratory Institute (CLSI) guidelines. Blood agar plates were inoculated with glycerol stocks of *E. coli* ATCC 25922, *K. pneumoniae* ATCC 13883, *A. baumannii* ATCC 17961, *P. aeruginosa* PAO1 and *S. aureus* USA300. The inoculated agar plates were then incubated for 16 h at 37 °C . Individually grown colonies were subsequently used to inoculate 5 mL aliquots of TSB that were then incubated at 37 °C with shaking at 220 rpm. *E. coli* 25922 MCR-1 glycerol stock was used to inoculate 5 mL of TSB supplemented with kanamycin that was then incubated for 16 h at 37 °C with shaking at 220 rpm. The next day the culture was diluted 100 fold in TSB supplemented with kanamycin and incubated at 37 °C with shaking at 220 rpm. In parallel, the lipopeptide antibiotics DMSO stocks to be assessed were serially diluted with MHB in polypropylene 96-well plates (50 µL in each well). Colistin sulfate stocks were dissolved in water before being diluted with MHB. Aliquots of the inoculated TSB were incubated until an OD_{600} of around 0.5 was reached. The bacterial suspensions were then diluted with MHB (2 x 10^5 CFU mL^{-1}) and added to the microplates containing the test compounds (50 µL to each well). The well-plates were sealed with an adhesive membrane and after 18 h of incubation at 37 °C with shaking at 600 rpm, the wells were visually inspected for bacterial

growth. MIC values reported are based on three technical replicates and defined as the lowest concentration of the compound that prevented visible growth of bacteria.

V. Hemolytic assays

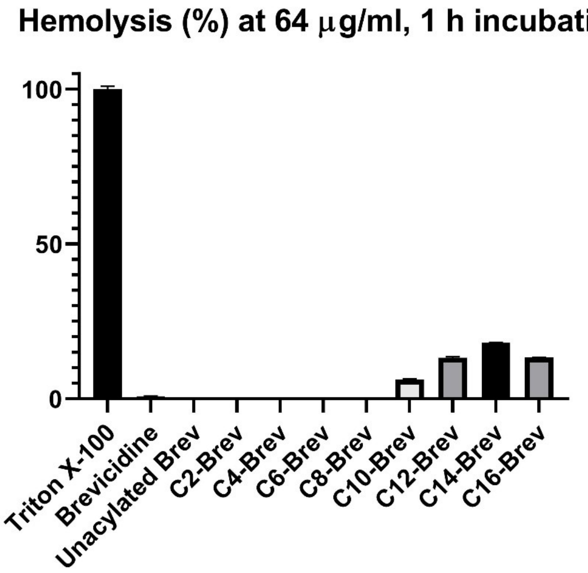

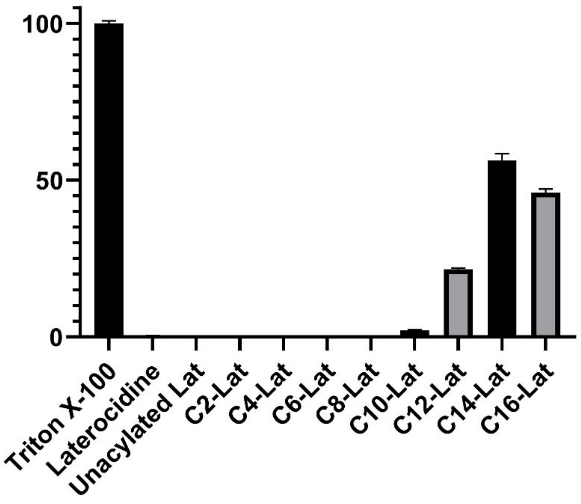

Experiments were performed in triplicate and Triton X-100 used as a positive control. Red blood cells from defibrinated sheep blood obtained from Thermo Fisher were centrifuged (400 g for 15 min at 4°C) and washed with Phosphate-Buffered Saline (PBS) containing 0.002% Tween20 (buffer) five times. Then, the red blood cells were normalized to obtain a positive control read-out between 2.5 and 3.0 at 415 nm to stay within the linear range with the maximum sensitivity. A serial dilution of the compounds (128 – 1 µg/mL, 75 µL) was prepared in a 96-well polypropylene plate. The outer border of the plate was filled with 75 µL buffer. Each plate contained a positive control (0.1% Triton-X final concentration, 75 µL) and a negative control (buffer, 75 µL) in triplicate. The normalized blood cells (75 µL) were added and the plates were incubated at 37 °C for 1 h while shaking at 500 rpm. A flat-bottom polystyrene plate with 100 µL buffer in each well was prepared. After incubation, the plates were centrifuged (800 g for 5 min at room temperature) and 25 µL of the supernatant was transferred to their respective wells in the flat-bottom plate. The values obtained from a read-out at 415 nm were corrected for background (negative control) and transformed to a percentage relative to the positive control.

VI. HPLC and HRMS analysis of peptides

Peptide	Name	Chemical	Calcd	Mass found	Calcd	Overall

		Formula	Exact Mass			Yield [%]
**1**	Brevicidine	$C_{74}H_{106}N_{18}O_{17}$	1518.7983	507.2688 $[M+3H]^{3+}$	507.2734	9
**9**	H-Brev	$C_{67}H_{94}N_{18}O_{16}$	1406.7095	704.3672 $[M+2H]^{2+}$	704.3620	2
**10**	C2-Brev	$C_{69}H_{96}N_{18}O_{17}$	1448.7201	1449.7290 $[M+H]^{+}$	1449.7274	3
**11**	C4-Brev	$C_{71}H_{100}N_{18}O_{17}$	1476.7514	1477.7579 $[M+H]^{+}$	1477.7592	25
**12**	C6-Brev	$C_{73}H_{104}N_{18}O_{17}$	1504.7827	1505.8070 $[M+H]^{+}$	1505.7905	19
**13**	C8-Brev	$C_{75}H_{108}N_{18}O_{17}$	1532.8140	1533.8483 $[M+H]^{+}$	1533.8218	4
**14**	C10-Brev	$C_{77}H_{112}N_{18}O_{17}$	1560.8453	521.2809 $[M+3H]^{3+}$	521.2809	4
**15**	C12-Brev	$C_{79}H_{116}N_{18}O_{17}$	1588.8766	795.4525 $[M+2H]^{2+}$	795.4456	3
**16**	C14-Brev	$C_{81}H_{120}N_{18}O_{17}$	1616.9079	809.4640 $[M+2H]^{2+}$	809.4612	2
**17**	C16-Brev	$C_{83}H_{124}N_{18}O_{17}$	1644.9392	823.4837 $[M+2H]^{2+}$	823.4769	4
**2**	Laterocidine	$C_{78}H_{113}N_{19}O_{18}$	1603.8511	802.9326 $[M+2H]^{2+}$	802.9329	2
**18**	H-Lat	$C_{69}H_{97}N_{19}O_{17}$	1463.7310	732.8731 $[M+2H]^{2+}$	732.8728	2
**19**	C2-Lat	$C_{71}H_{99}N_{19}O_{18}$	1505.7415	753.8783 $[M+2H]^{2+}$	753.8781	1
**20**	C4-Lat	$C_{73}H_{103}N_{19}O_{18}$	1533.7728	767.8944 $[M+2H]^{2+}$	767.8937	3
**21**	C6-Lat	$C_{75}H_{107}N_{19}O_{18}$	1561.8041	781.9097 $[M+2H]^{2+}$	781.9094	2
**22**	C8-Lat	$C_{77}H_{111}N_{19}O_{18}$	1589.8354	795.9254 $[M+2H]^{2+}$	795.9250	2
**23**	C10-Lat	$C_{79}H_{115}N_{19}O_{18}$	1617.8667	809.9410 $[M+2H]^{2+}$	809.9407	3
**24**	C12-Lat	$C_{81}H_{119}N_{19}O_{18}$	1645.8980	823.9567 $[M+2H]^{2+}$	823.9563	0.4
**25**	C14-Lat	$C_{83}H_{123}N_{19}O_{18}$	1673.9293	851.9881 $[M+2H]^{2+}$	851.9876	1
**26**	C16-Lat	$C_{85}H_{127}N_{19}O_{18}$	1701.9606	837.9722 $[M+2H]^{2+}$	837.9720	1

Table S1. Peptide number, name, chemical formula, exact mass, mass found and overall yield for peptides **1** – **26**.

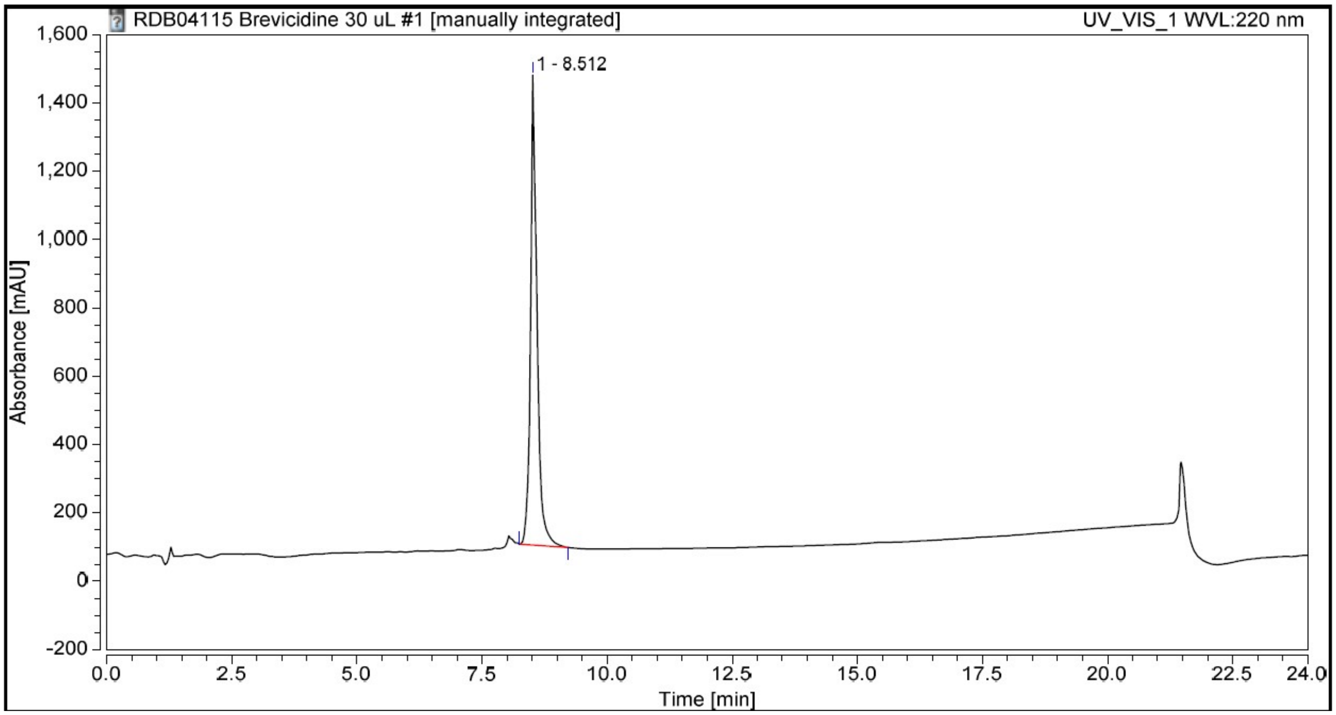

Fig. S1. HPLC trace showing the reinjection of purified brevicidine (**1**). The peptide eluted as a single peak at 8.512 min using the HPLC **method C** outlined in part III.

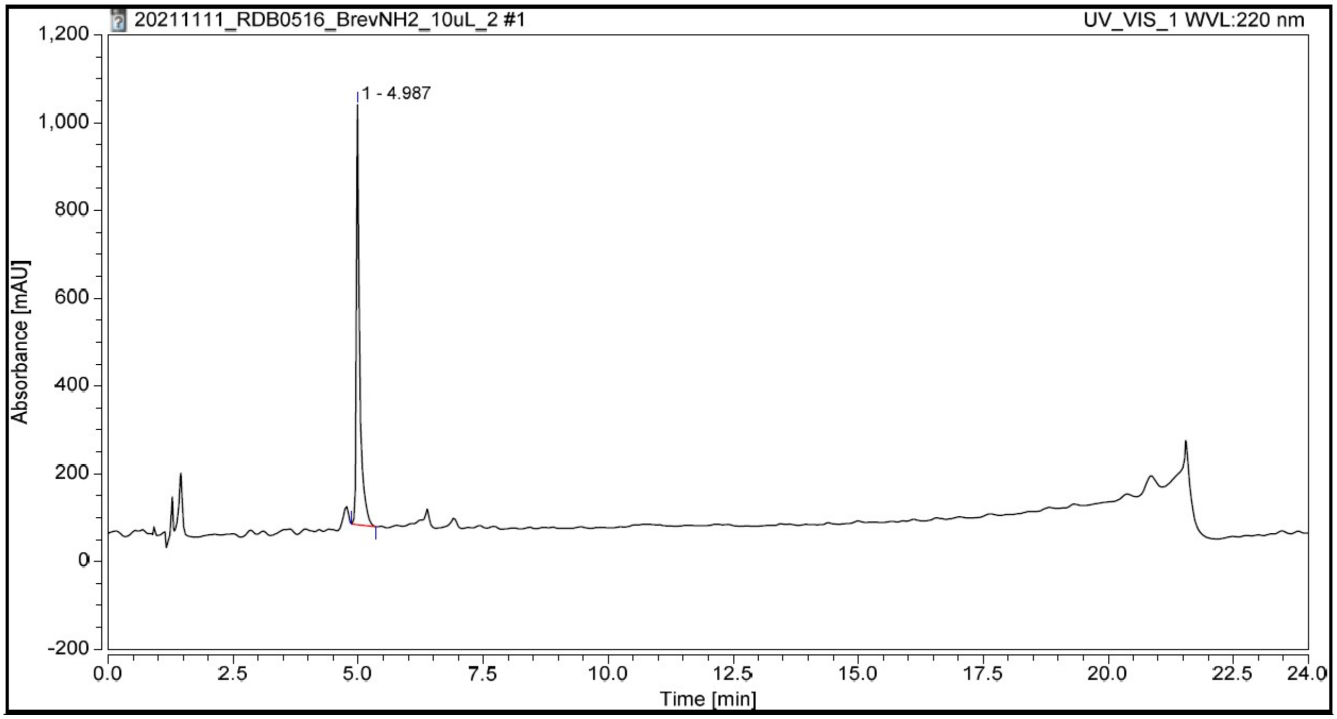

Fig. S2. HPLC trace showing the reinjection of purified unacylated brevicidine (**9**). The peptide eluted as a single peak at 4.987 min using the HPLC **method C** outlined in part III.

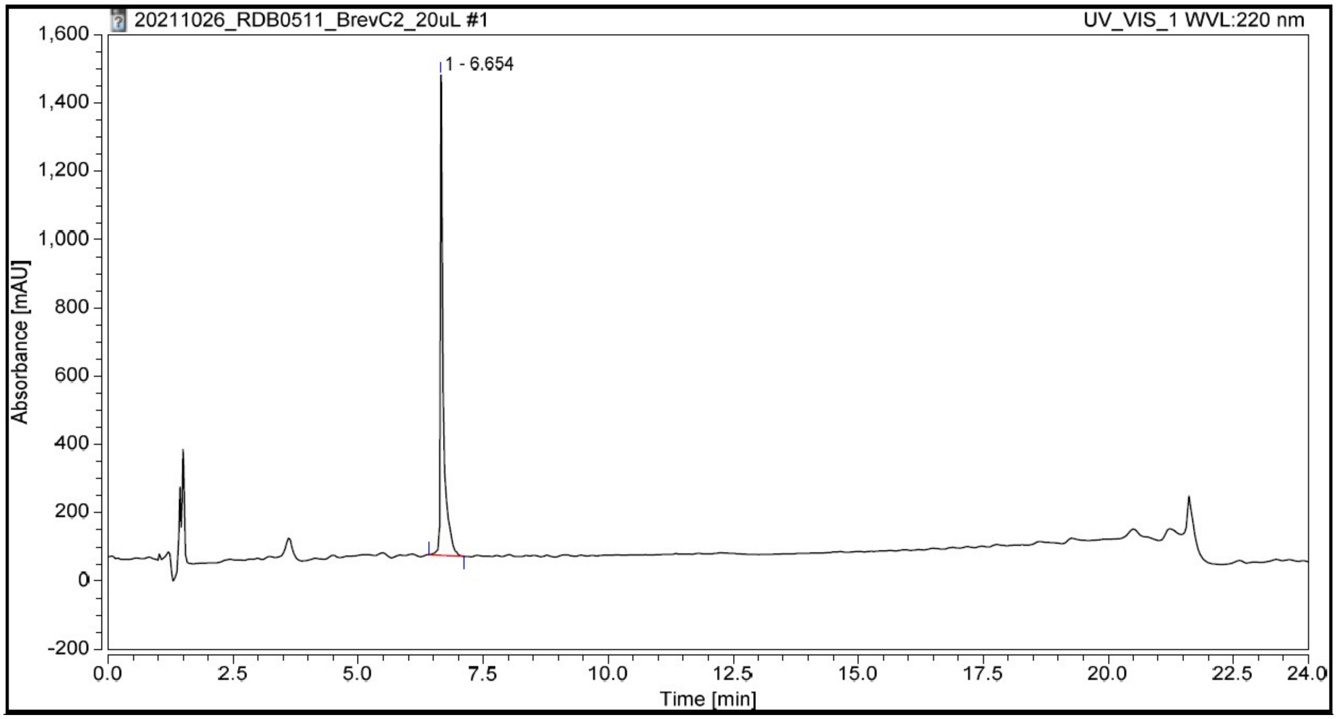

Fig. S3. HPLC trace showing the reinjection of purified C2-Brev (**10**). The peptide eluted as a single peak at 6.654 min using the HPLC **method C** outlined in part III.

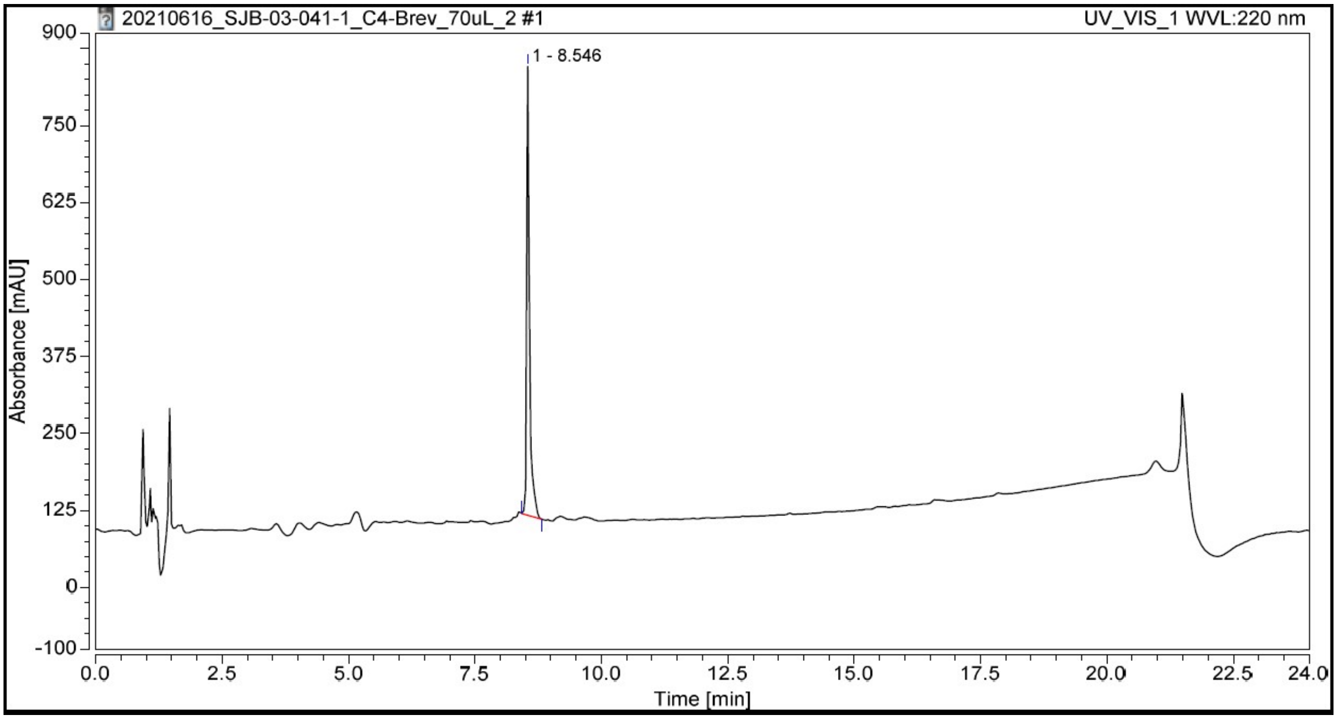

Fig. S4. HPLC trace showing the reinjection of purified C4-Brev (**11**). The peptide eluted as a single peak at 8.546 min using the HPLC **method C** outlined in part III.

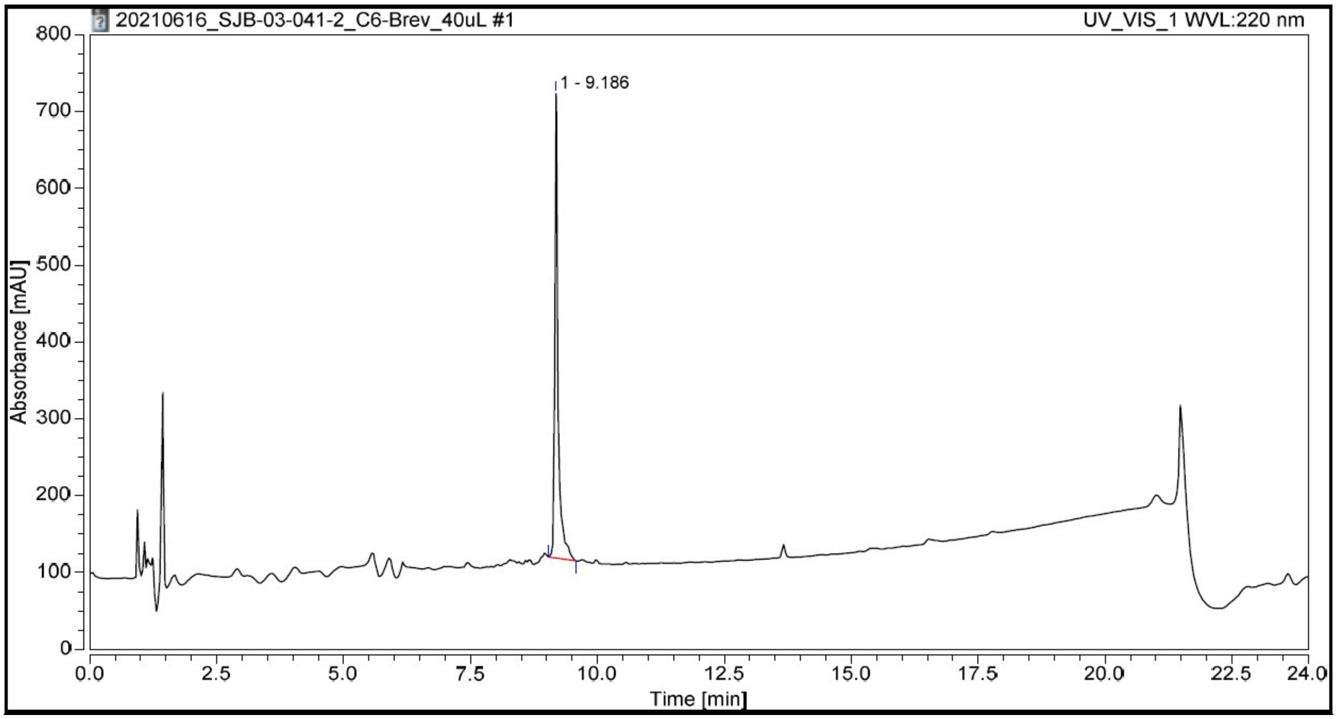

Fig. S5. HPLC trace showing the reinjection of purified C6-Brev (**12**). The peptide eluted as a single peak at 9.186 min using the HPLC **method C** outlined in part III.

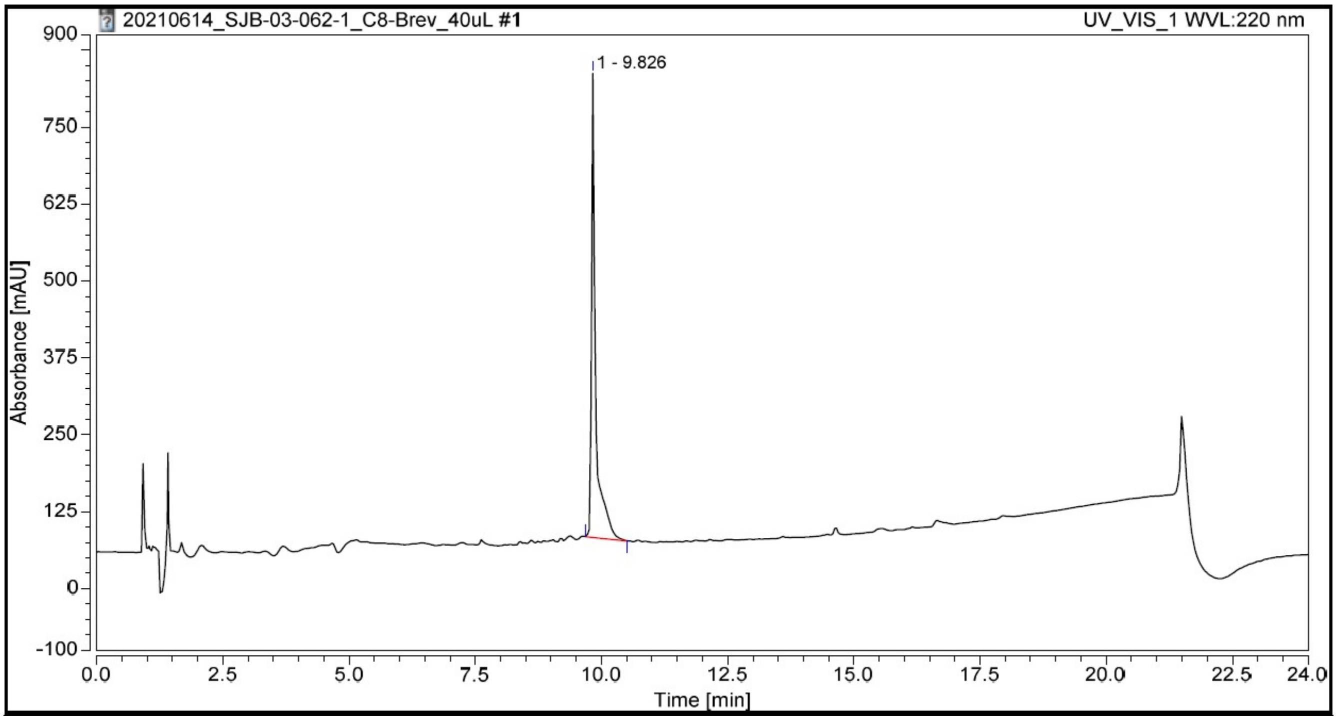

Fig. S6. HPLC trace showing the reinjection of purified C8-Brev (**13**). The peptide eluted as a single peak at 9.826 min using the HPLC **method C** outlined in part III.

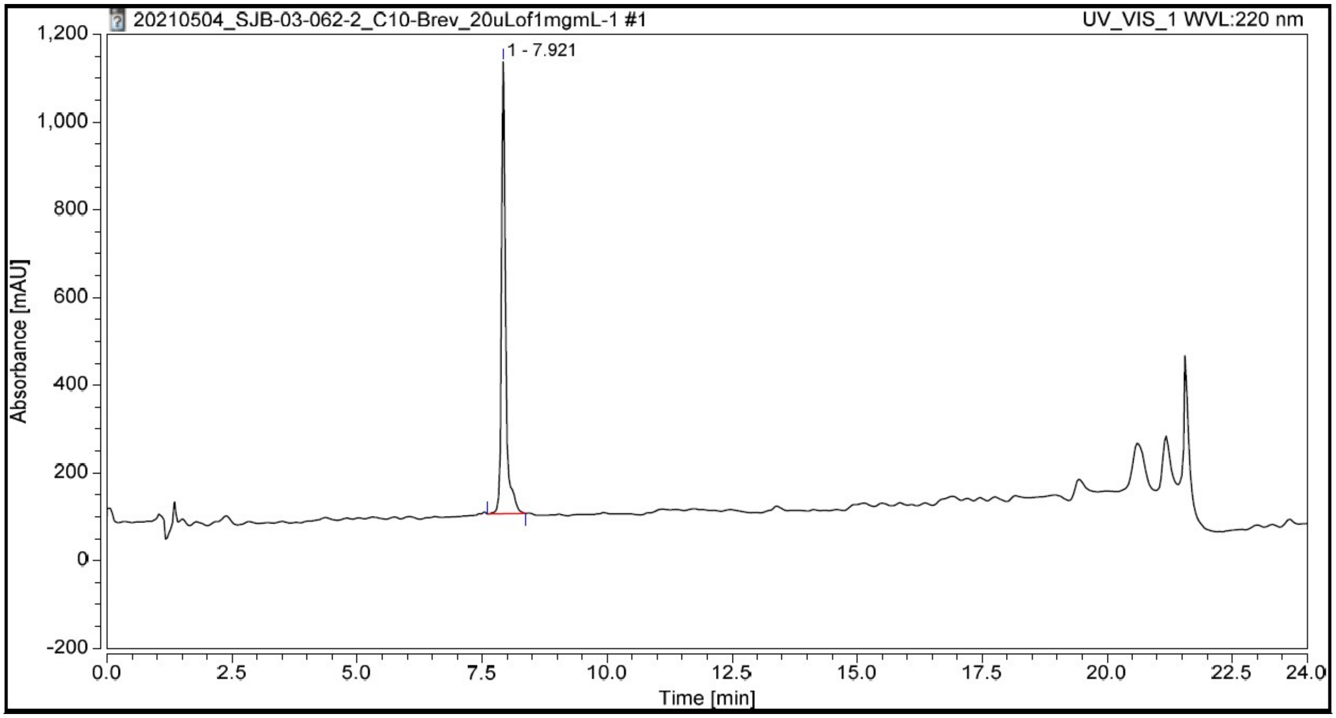

Fig. S7. HPLC trace showing the reinjection of purified C10-Brev (**14**). The peptide eluted as a single peak at 7.921 min using the HPLC **method C** outlined in part III.

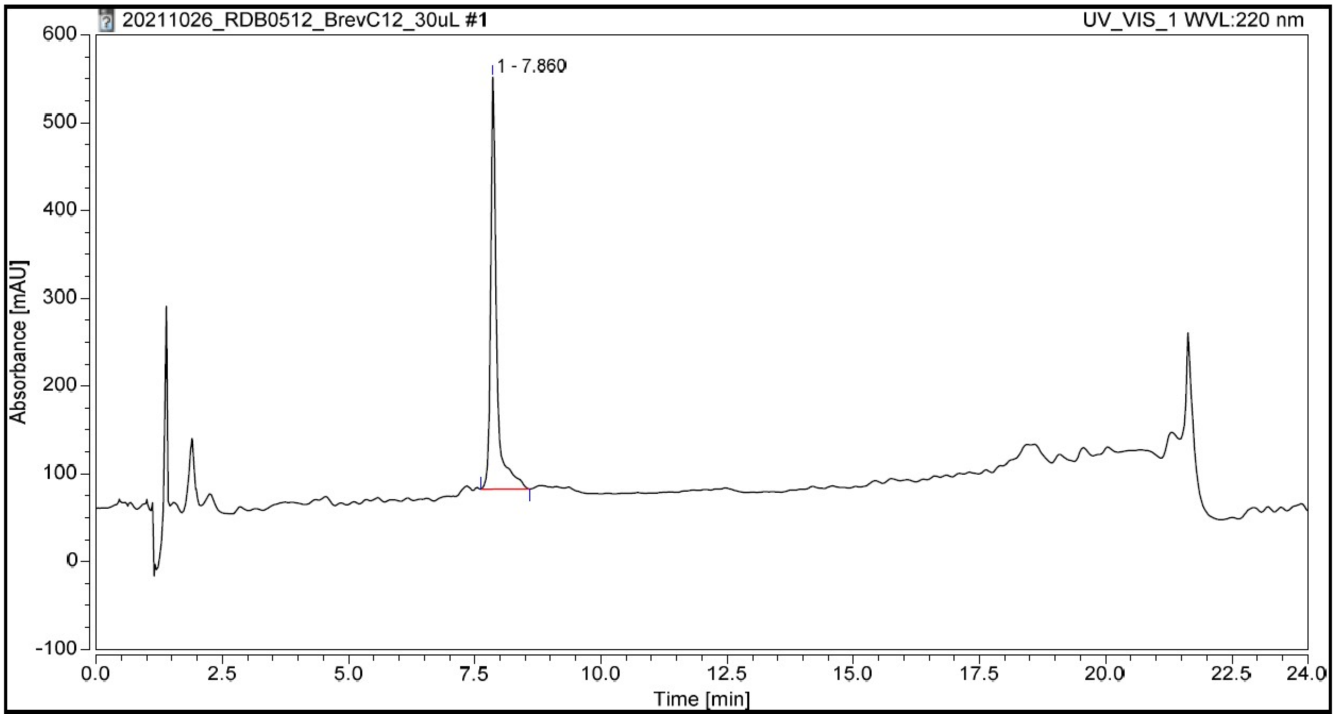

Fig. S8. HPLC trace showing the reinjection of purified C12-Brev (**15**). The peptide eluted as a single peak at 7.860 min using the HPLC **method C** outlined in part III.

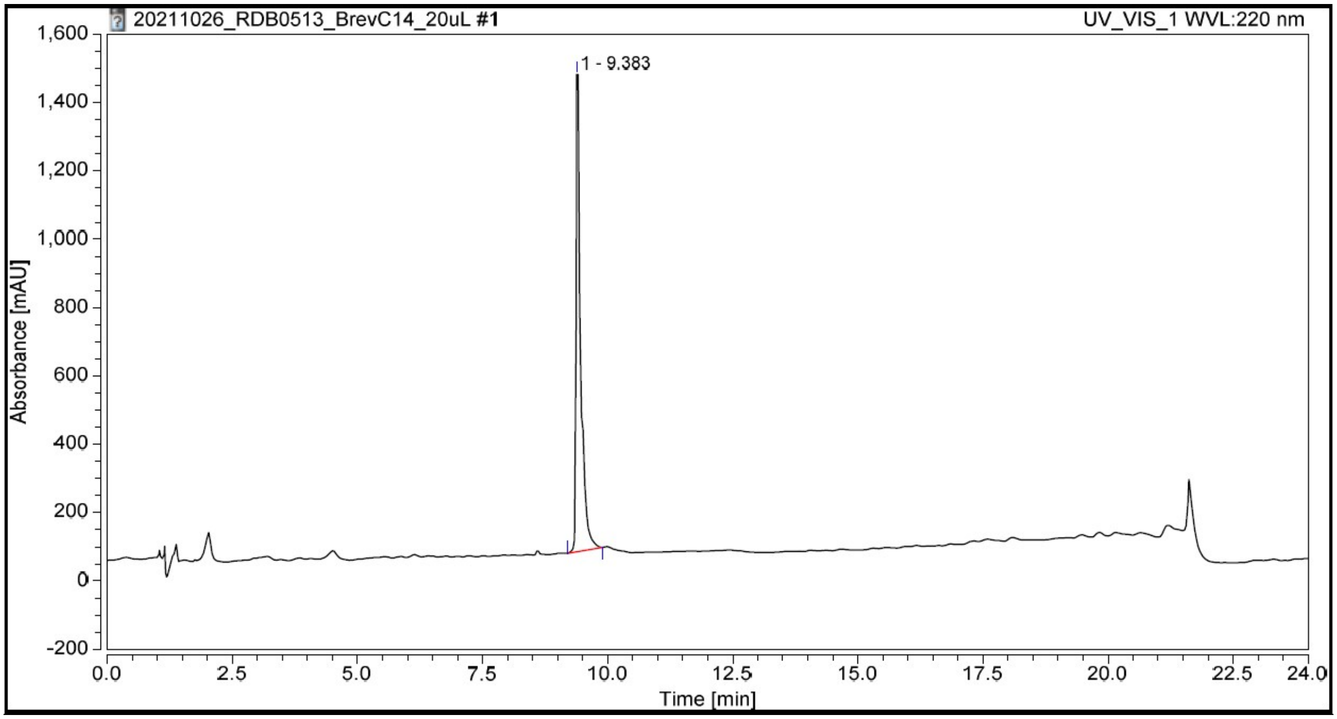

Fig. S9. HPLC trace showing the reinjection of purified C14-Brev (**16**). The peptide eluted as a single peak at 9.383 min using the HPLC **method C** outlined in part III.

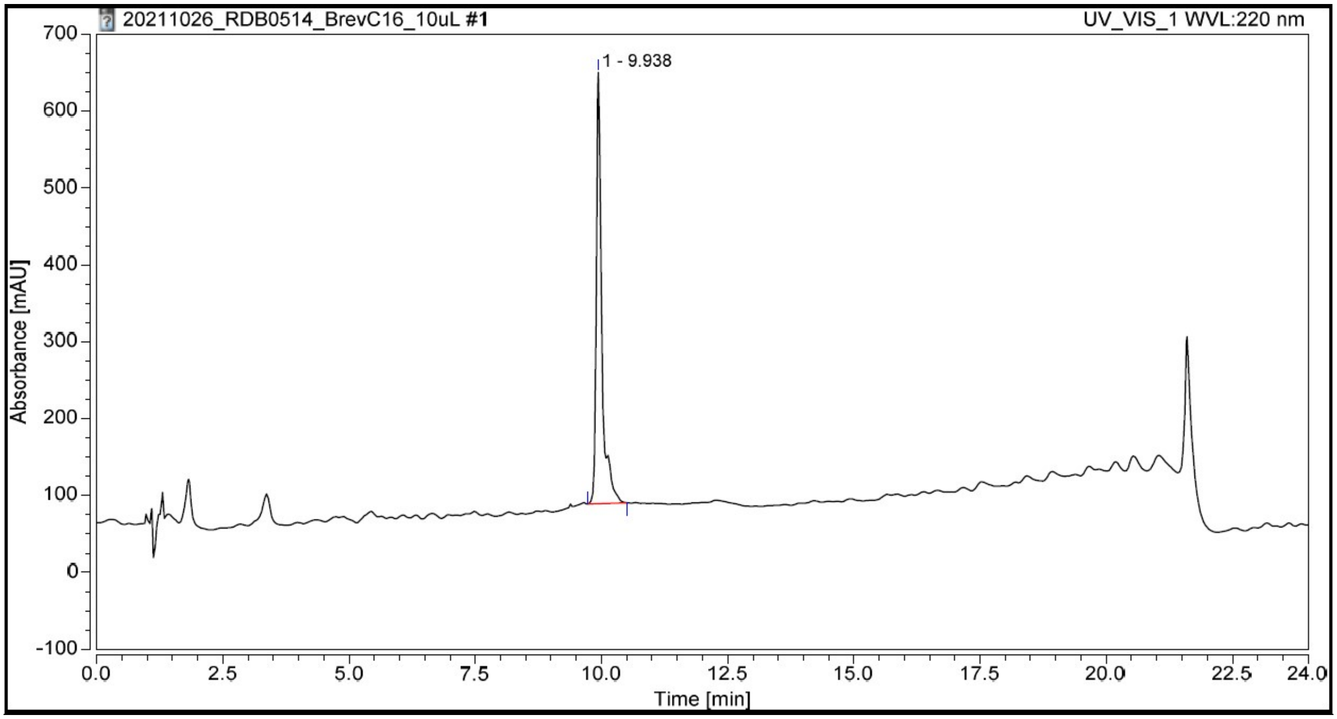

Fig. S10. HPLC trace showing the reinjection of purified C16-Brev (**17**). The peptide eluted as a single peak at 9.938 min using the HPLC **method C** outlined in part III.

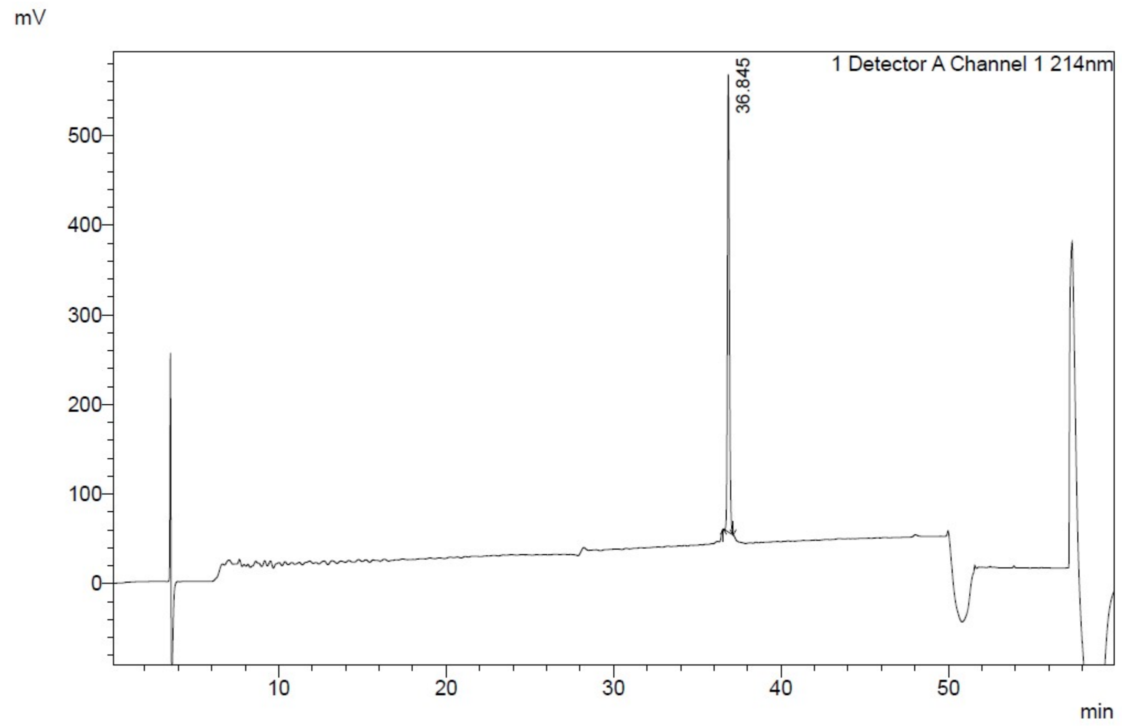

Fig. S11. HPLC trace showing the reinjection of purified laterocidine (**2**). The peptide eluted as a single peak at 36.85 min using the HPLC **method D** outlined in part III.

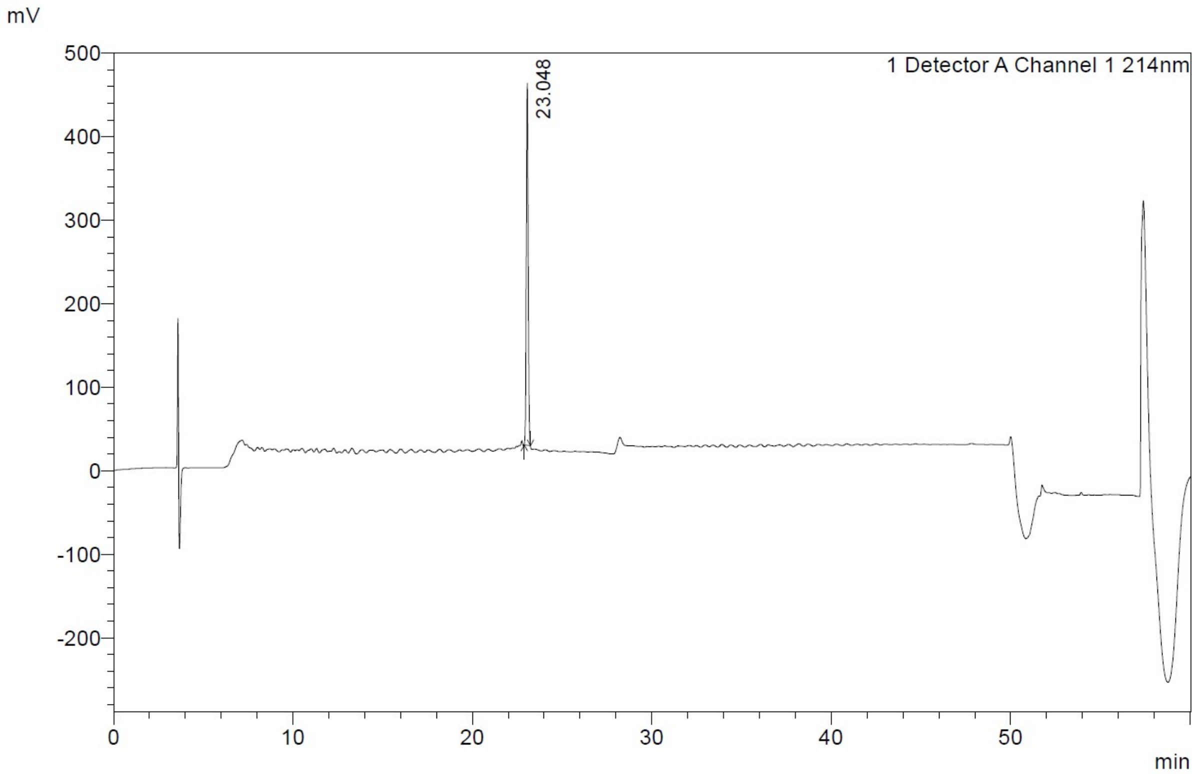

Fig. S12. HPLC trace showing the reinjection of purified unacylated laterocidine (**18**). The peptide eluted as a single peak at 23.05 min using the HPLC **method D** outlined in part III.

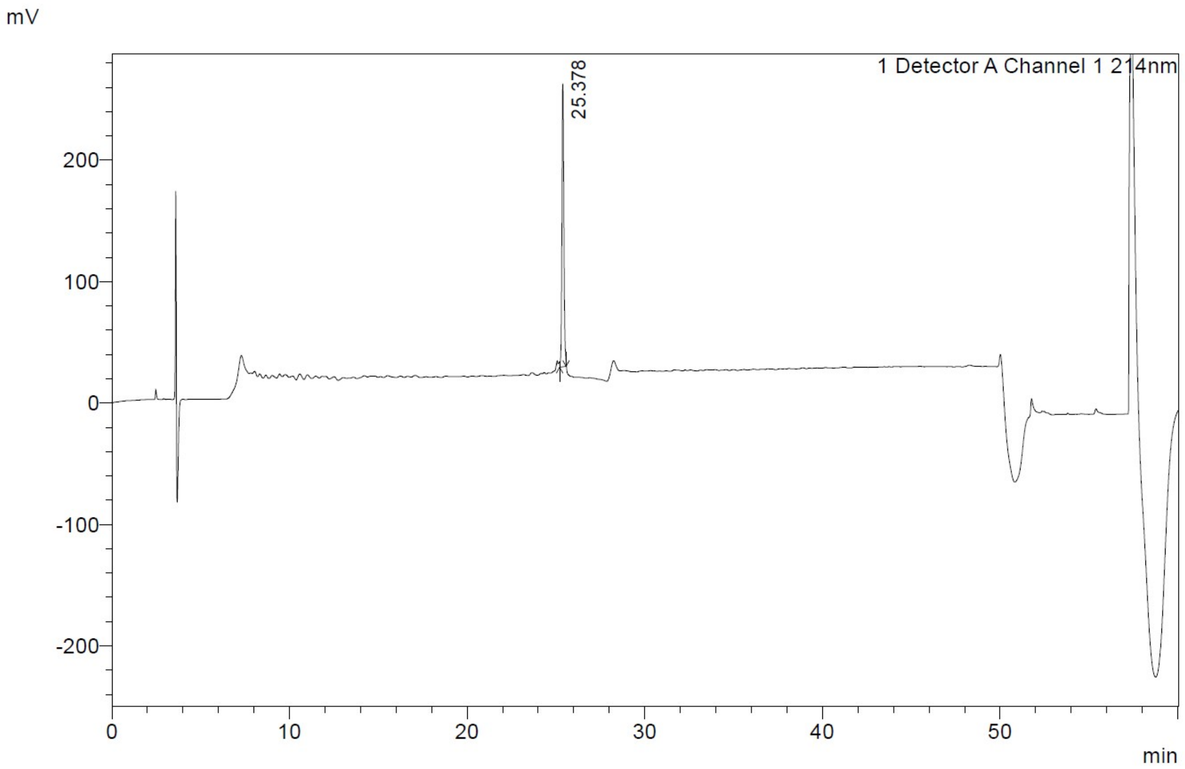

Fig. S13. HPLC trace showing the reinjection of purified C2-Lat (**19**). The peptide eluted as a single peak at 25.378 min using the HPLC **method D** outlined in part III.

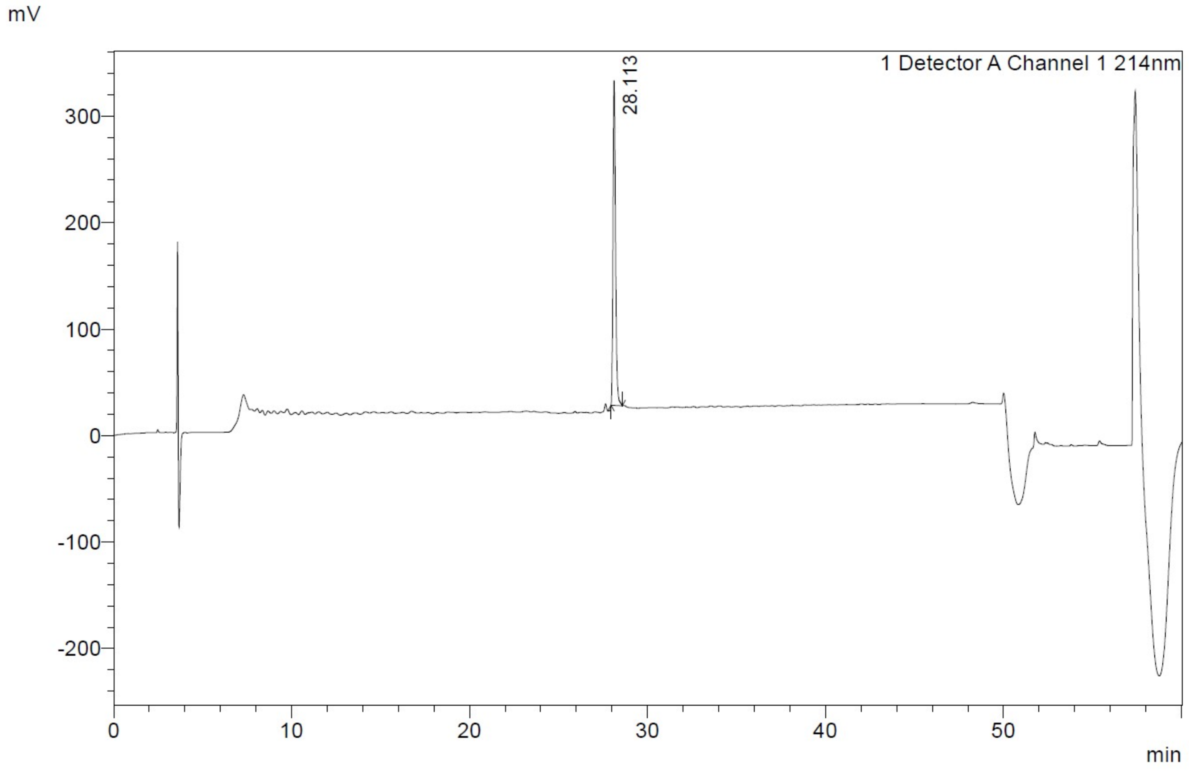

Fig. S14. HPLC trace showing the reinjection of purified C4-Lat (**20**). The peptide eluted as a single peak at 28.113 min using the HPLC **method D** outlined in part III.

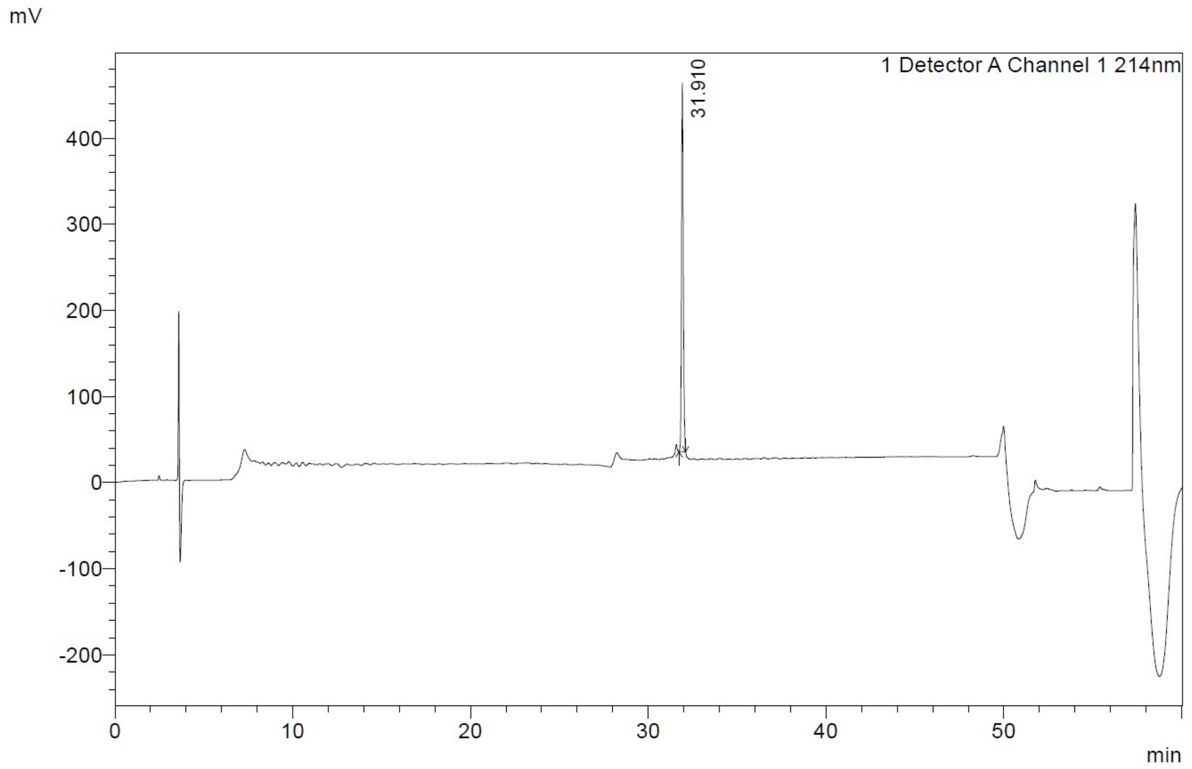

Fig. S15. HPLC trace showing the reinjection of purified C6-Lat (**21**). The peptide eluted as a single peak at 31.910 min using the HPLC **method D** outlined in part III.

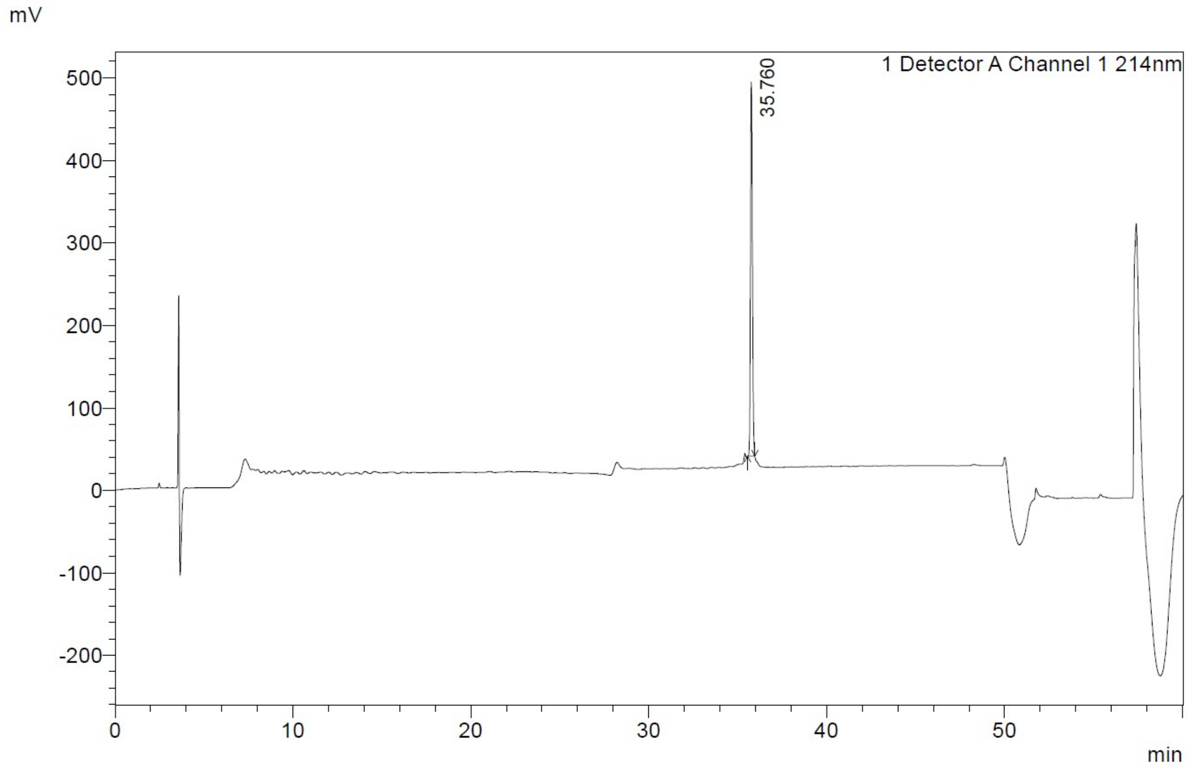

Fig. S16. HPLC trace showing the reinjection of purified C8-Lat (**22**). The peptide eluted as a single peak at 35.760 min using the HPLC **method D** outlined in part III.

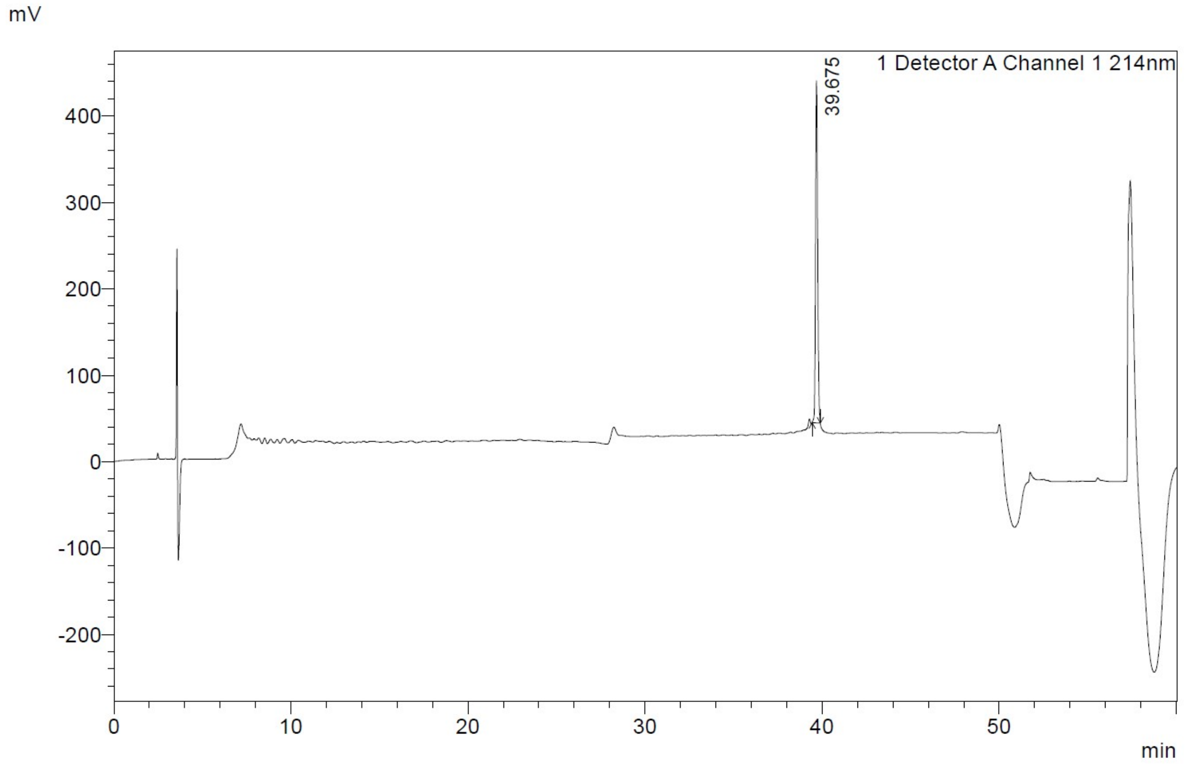

Fig. S17. HPLC trace showing the reinjection of purified C10-Lat (**23**). The peptide eluted as a single peak at 39.675 min using the HPLC **method D** outlined in part III.

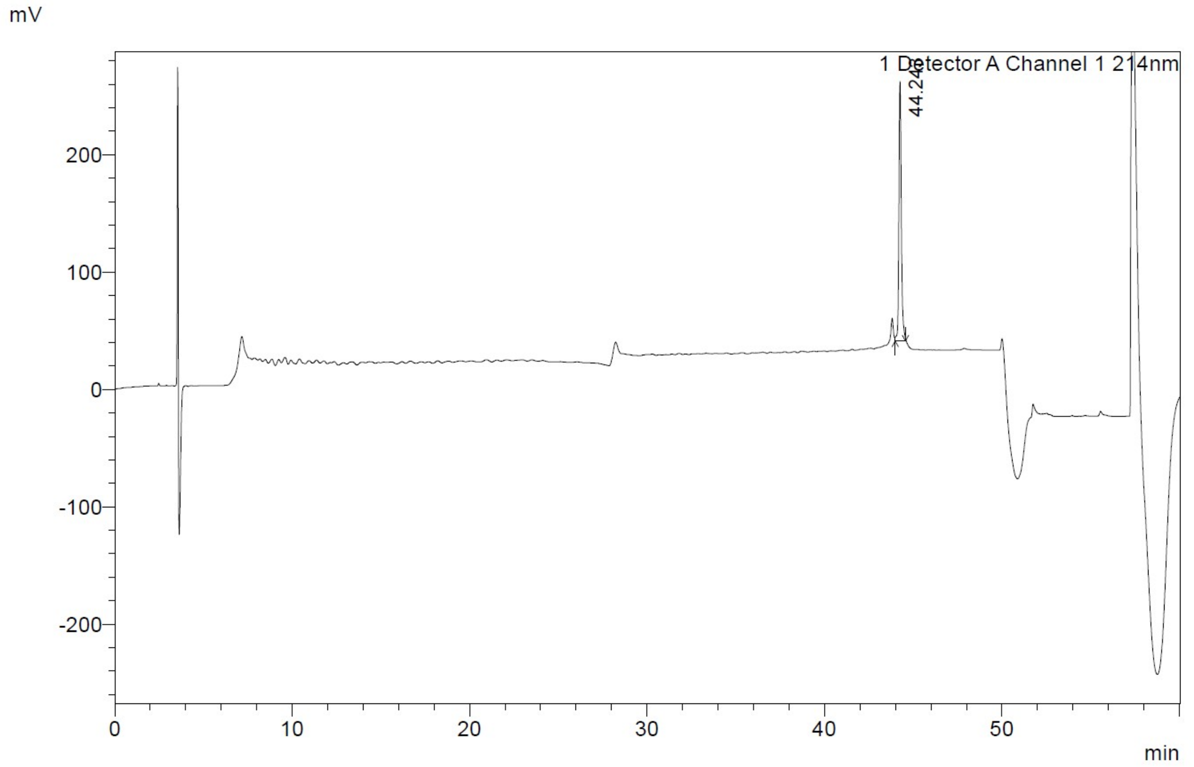

Fig. S18. HPLC trace showing the reinjection of purified C12-Lat (**24**). The peptide eluted as a single peak at 44.24 min using the HPLC **method D** outlined in part III.

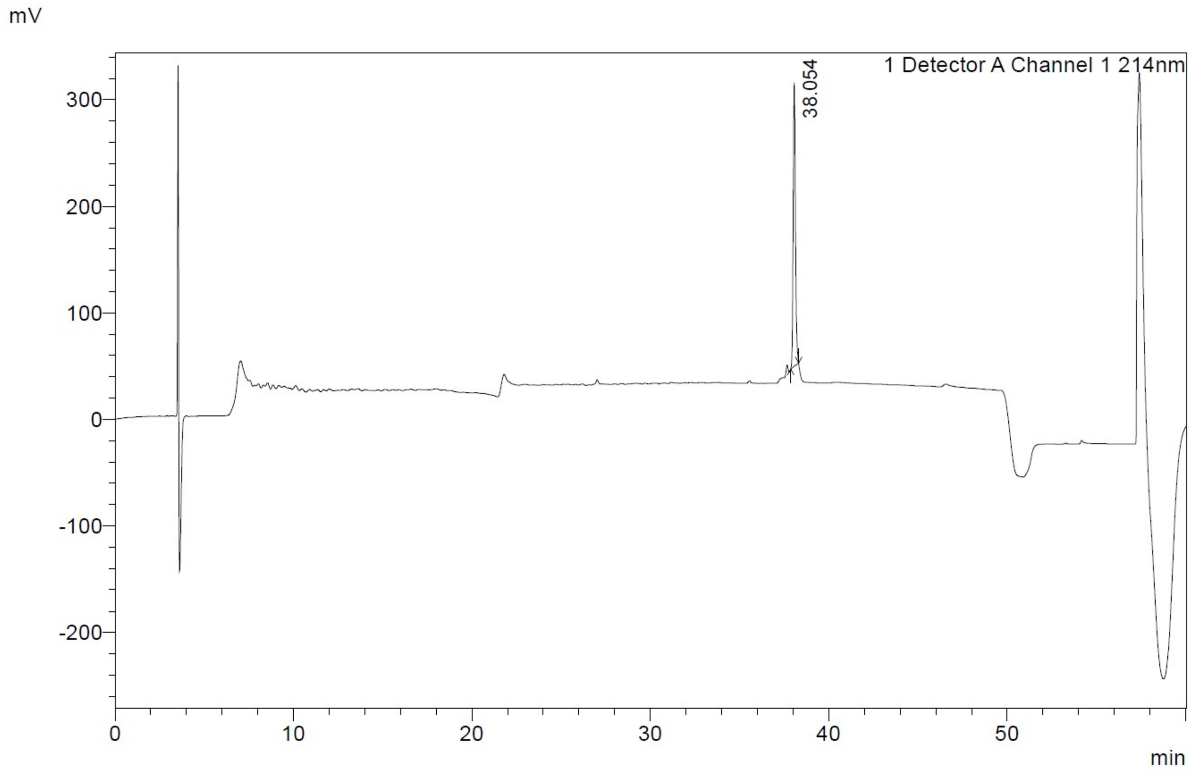

Fig. S19. HPLC trace showing the reinjection of purified C14-Lat (**25**). The peptide eluted as a single peak at 38.054 min using the HPLC **method D** outlined in part III.

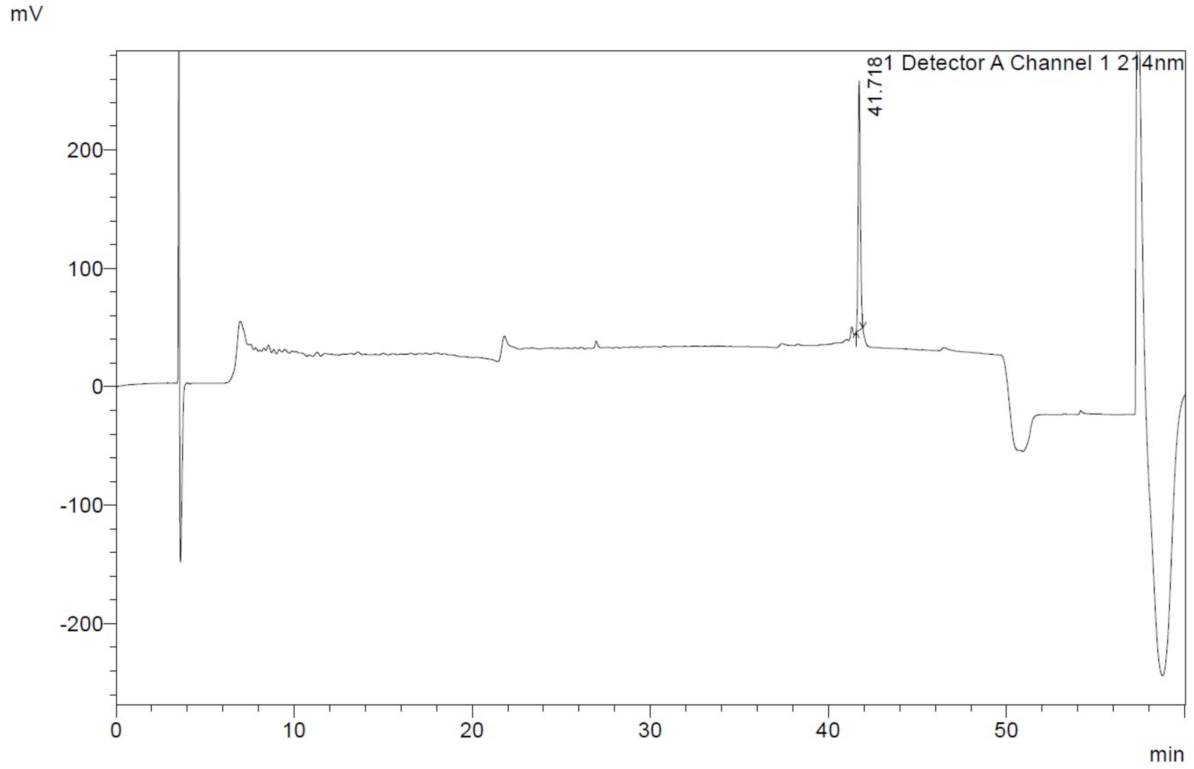

Fig. S20. HPLC trace showing the reinjection of purified C16-Lat (**26**). The peptide eluted as a single peak at 41.718 min using the HPLC **method D** outlined in part III.

VII. References

[1] S. Mukherjee, W. A. van der Donk, *J. Am. Chem. Soc.*, 2014, **136**, 10450-10459.

[2] J. Ge, L. Li, S. Q. Yao, *Chem Commun.*, 2011, **47**, 10939-10941.

[3] f. Freire, J. D. Fisk, A. J. Peopls, M. Ivancic, I. A. Guzei, S. H. Gellman, *J. Am. Chem. Soc.*, 2008, **130**, 7839-7841.

[4] K. Al-Ayed, R. D. Ballantine, M. Hoekstra, S. J. Bann, C. M. J. Wesseling, A. T. Bakker, Z. Zhong, Y. X. Li, N. C. Brüchle, M. van der Stelt, S. A. Cochrane and N. I. Martin, *Chem. Sci.*, 2022, **13**, 3563-3570.

[5] M. Gude, J. Ryf, P. D. White, *Lett. Pept. Sci.*, 2002, **9**, 203-206.
